# Variability of allergenicity within 29 genotypes including some genetically modified genotypes

**DOI:** 10.1186/2045-7022-3-S3-P91

**Published:** 2013-07-25

**Authors:** R Lupi, S Masci, F Pineau, S Denery-Papini, C Larré

**Affiliations:** 1 BIA-ALL, INRA, Nantes, France; 2 DAFNE, Università degli Studi della Tuscia, Viterbo, Italy

## Background

Breeding of cereals has been particularly active in the last hundred years, resulting in spectacular improvements in agronomic, resistance or technological traits. Unfortunately for predisposed consumers wheat may induce allergenicity either food or respiratory. More recently, the genetic modification (GM) technology for crop improvement has emerged and although GM wheats are not cultivated at the moment, it is not excluded that they are being produced. In the frame of GM evaluation and according to the codex Alimentarius, the allergenicity has to be evaluated.

In this work we intend to measure the allergenicity of GM wheat lines, their parents and to compare it to a set of cultivated varieties for which the “history of safe use” is not or poorly documented.

## Methods

The studied genotypes belong to the two main wheat cultivated species, T. aestivum and T. Durum. In this study, IgE were collected from patients suffering from food or respiratory allergy and used as probes to detect wheat flour allergens in an ELISA assay. Data were analysed using paired samples t-test and two-way ANOVA. In order to increase the sensibility the salt soluble proteins fraction was separated into two fractions, metabolic and chloroform-methanol soluble (CM), their protein composition was analysed and their IgE reactivity controlled.

## Results

Both fractions were IgE reactive and the CM-like fraction was enriched in alpha amylase inhibitors and LTP. Two “factors” were studied, genotype and serum, both have a significant effect on the IgE-binding capacity. The comparison of means revealed significant differences in the IgE concentration for 3 GM lines versus their wild type whatever the fraction considered. However, it was noticeable that significant differences were also detected between cultivars.

The reactivity of the T. durum cultivars were in the range of 28-50 ng/ml and 40-70 ng/ml for the metabolic and CM_like fractions respectively. That of GM lines, even those that were significantly different from their wild type lines, were included in these reactivity ranges. Similar results were obtained for T. aestivum cultivars.

## Conclusion

This study drives us to conclude that the transformation impacts the allergenicity, by increasing or decreasing it. The level of allergenicity measured for the GM lines was always lower that the higher value measured for the cultivated genotypes tested.

## Disclosure of interest

None declared.

